# NeuroBridge ontology: computable provenance metadata to give the long tail of neuroimaging data a FAIR chance for secondary use

**DOI:** 10.3389/fninf.2023.1216443

**Published:** 2023-07-24

**Authors:** Satya S. Sahoo, Matthew D. Turner, Lei Wang, Jose Luis Ambite, Abhishek Appaji, Arcot Rajasekar, Howard M. Lander, Yue Wang, Jessica A. Turner

**Affiliations:** ^1^Case Western Reserve University, Cleveland, OH, United States; ^2^Department of Psychiatry and Behavioral Health, The Ohio State University Wexner Medical Center, Columbus, OH, United States; ^3^University of Southern California, Los Angeles, CA, United States; ^4^BMS College of Engineering, Bengaluru, India; ^5^University of North Carolina at Chapel Hill, Chapel Hill, NC, United States

**Keywords:** FAIR neuroimaging data, computable provenance metadata, NeuroBridge ontology, ontology text annotation, W3C PROV ontology

## Abstract

**Background:**

Despite the efforts of the neuroscience community, there are many published neuroimaging studies with data that are still not *findable* or *accessible*. Users face significant challenges in *reusing* neuroimaging data due to the lack of provenance metadata, such as experimental protocols, study instruments, and details about the study participants, which is also required for *interoperability.* To implement the FAIR guidelines for neuroimaging data, we have developed an iterative ontology engineering process and used it to create the NeuroBridge ontology. The NeuroBridge ontology is a computable model of provenance terms to implement FAIR principles and together with an international effort to annotate full text articles with ontology terms, the ontology enables users to locate relevant neuroimaging datasets.

**Methods:**

Building on our previous work in metadata modeling, and in concert with an initial annotation of a representative corpus, we modeled diagnosis terms (e.g., schizophrenia, alcohol usage disorder), magnetic resonance imaging (MRI) scan types (T1-weighted, task-based, etc.), clinical symptom assessments (PANSS, AUDIT), and a variety of other assessments. We used the feedback of the annotation team to identify missing metadata terms, which were added to the NeuroBridge ontology, and we restructured the ontology to support both the final annotation of the corpus of neuroimaging articles by a second, independent set of annotators, as well as the functionalities of the NeuroBridge search portal for neuroimaging datasets.

**Results:**

The NeuroBridge ontology consists of 660 classes with 49 properties with 3,200 axioms. The ontology includes mappings to existing ontologies, enabling the NeuroBridge ontology to be interoperable with other domain specific terminological systems. Using the ontology, we annotated 186 neuroimaging full-text articles describing the participant types, scanning, clinical and cognitive assessments.

**Conclusion:**

The NeuroBridge ontology is the first computable metadata model that represents the types of data available in recent neuroimaging studies in schizophrenia and substance use disorders research; it can be extended to include more granular terms as needed. This metadata ontology is expected to form the computational foundation to help both investigators to make their data FAIR compliant and support users to conduct reproducible neuroimaging research.

## 1. Introduction

Reproducible science involving replication and reproducibility using meta-analysis as well as mega-analyses are critical to the advancement of neuroimaging research (Dinov et al., 2010; Poldrack et al., 2017; Kennedy et al., 2019). Reanalysis of a study, either with alternate analyses of the original experiment or with novel analyses that conform to the data is relatively easy if the original data and the associated provenance metadata are available to other researchers (Sahoo et al., 2019; Huber et al., 2020). Well-designed mega- and meta-analyses require the identification of studies that use experimental methods and subjects that are similar or equivalent to the original study; therefore, provenance metadata that describes this contextual information is critical for the identification and harnessing of data from existing studies for rigorous replication. The Findable, Accessible, Interoperable, and Reusable (FAIR) guiding principles adopted in 2014 aim to facilitate the discoverability and accessibility of the useful datasets (Wilkinson et al., 2016). However, concrete implementation of the FAIR guiding principles has been a key challenge (Musen et al., 2022), especially for neuroimaging databases and repositories [The National Institute of Mental Health Data Archive (NDA), 2023], which are often stored in silos with limited support for FAIR principles.

For example, the neuroimaging data repositories supported by different divisions within the US National Institutes of Health (NIH) lack common terminology and representation format for metadata information describing the datasets [The National Institute of Mental Health Data Archive (NDA), 2023]. Similarly, the large volume of neuroimaging datasets that are collected in hundreds of laboratories around the world each year are only described in journal publications without being made accessible through organized data management systems (Sejnowski et al., 2014). These underutilized data form the “long tail of science” (Ferguson et al., 2014; Frégnac, 2017), and finding these datasets requires tedious search of published literature for relevant neuroimaging studies through manual review of papers to extract the provenance metadata of the studies. The metadata terms describe the structure and methods used in the study, such as the profile of the participants recruited for the study (e.g., patients with schizophrenia, cocaine users and their family members), the type of neuroimaging data collected [e.g., T1-weighted imaging, task-based functional magnetic resonance imaging (fMRI)], and the clinical and cognitive assessment instruments used in the study (e.g., SAPS/SANS, RAVLT, AUDIT).

PubMed and Google Scholar search features allow users to find papers related to a neuroimaging question of interest; however, the results do not analyze the study metadata such as the experimental design, the modality of data collected, and the status of data sharing. These existing search engines are powerful tools for exhaustive search using sophisticated artificial intelligence methods to find relevant results; however, the lack of support for FAIR principles makes it difficult for users to find relevant papers with accessible study data. To address this limitation, we are developing the NeuroBridge data discovery platform as part of the NIH-funded Collaborative Research in Computational Neuroscience (CRCNS) program to be a bridge between neuroimaging researchers and the relevant data published in literature. The NeuroBridge platform aims to identify, index, and analyze provenance metadata information from neuroimaging articles available in the PubMed Central repository and map specific studies to user queries related to research hypotheses. The NeuroBridge platform with its multiple components and sources is described in more detail in a companion paper in this Research Topic (Wang et al., Under Review). To enable the modeling of computable metadata that underpins the data search platform, we developed the NeuroBridge ontology based on FAIR guidelines for the neuroimaging domain.

### 1.1. Standardized provenance for implementing FAIR principles

The FAIR principles have been widely endorsed by funding agencies, including the NIH, individual researchers, and data curators to facilitate open science and maximize the reusability of existing resources. However, the lack of standardized metadata models that can be used by users in a specific domain to implement the FAIR principles and make their datasets FAIR compatible has been noted by recent studies (Musen et al., 2022). It is difficult for investigators to: (1) enumerate the relevant metadata terms that are necessary for understanding the experiment details that generated a dataset, which will ensure that the dataset can be reused either as part of a meta-analysis or new study; and (2) encode the relevant metadata terms in a machine interpretable standard format. In our earlier work in the field of data sharing in neurological disorders such as epilepsy and sleep disorder, we developed a metadata framework that classified provenance metadata related to research studies into the three categories of *study instrument*, *study data*, and *study method* (called the S3 model) as part of the Provenance for Clinical and Health Research (ProvCaRe) project (Sahoo et al., 2019). The S3 model is built on many existing reproducibility focused metadata guidelines such as the Consolidated Standards of Reporting Trials (CONSORT) guidelines (Schulz et al., 2010), the Animals in Research: Reporting *In Vivo* Experiments (ARRIVE) guidelines (Kilkenny et al., 2010), and the Problem/Population, Intervention, Comparison, Outcome and Time (PICOT) model (Richardson et al., 1995), among other guidelines.

The S3 model was formalized into a computable, machine interpretable format called the ProvCaRe ontology, which extended the World Wide Web Consortium (W3C) PROV specification to represent provenance metadata for biomedical domain. The PROV specification was developed as a standard provenance model for cross-domain interoperability and has been widely used to support FAIR guidelines in a variety of applications (Richardson et al., 1995; Poldrack and Gorgolewski, 2014). The PROV ontology formalized the PROV terms in an ontology using the description logic-based Web Ontology Language (OWL) with built-in extensibility features, which was used to create the ProvCaRe ontology for the broad biomedical domain. The NeuroBridge ontology is built on the same PROV specifications, and it is focused on the neuroimaging domain to support sharing and secondary use of experiment data.

### 1.2. Related work and the NeuroBridge project

There has long been a recognition of the importance of data sharing in neuroimaging studies and there has been multiple efforts to standardize terminologies describing neuroimagin datasets (Poldrack and Gorgolewski, 2014). We have contributed to or developed multiple projects to formalize aspects of these terminologies, for example neuroanatomical concepts in the Neuroscience Information Framework (NIF) project (Imam et al., 2012), the cognitive processes and measures (CogAtlas) project (Turner et al., 2011), details of the behavioral experiments used in functional neuroimaging (Cognitive Paradigm Ontology) (Turner and Laird, 2012), and the neuroimaging data analysis (NIDM ontology) (Maumet et al., 2016). Although these previous projects include model terms related to various aspects involved in neuroimaging studies, they lack provenance metadata terms at the appropriate level of granularity to describe the clinical or cognitive instruments used, the types of neuroimaging data collected, and information about the groups of study participants. The NeuroBridge ontology addresses this gap for neuroimaging studies.

The NeuroBridge platform overall builds closely on our work done in the SchizConnect project, which was developed to access multiple institutional neuroimaging databases (Wang et al., 2016). The SchizConnect project allowed a researcher to query for datasets that were relevant to their study hypothesis regarding schizophrenia, for example, a query for datasets including individuals with a diagnosis of schizophrenia, male, over 35 years old, and with a resting state fMRI scan on a 3T scanner. In response to this user query, the SchizConnect platform returned the data matching the query criteria from the different studies indexed by the platform to the user for download and analysis. The development of the SchizConnect platform involved the creation of a terminology, a usable subset of terms to describe neuroimaging datasets, which were informed in part by users and in part by the Organization for Human Brain Mapping (OHBM) Committee on Best Practice in Data Analysis and Sharing (COBIDAS) for reporting fMRI studies databases (Turner et al., 2015). The SchizConnect terminology consisted of terms to describe the different types of schizophrenia groups included in the available studies, the imaging types, the scanner information and the other attendant clinical, cognitive, or behavioral data that were part of the SchizConnect database.

**FIGURE 1:**
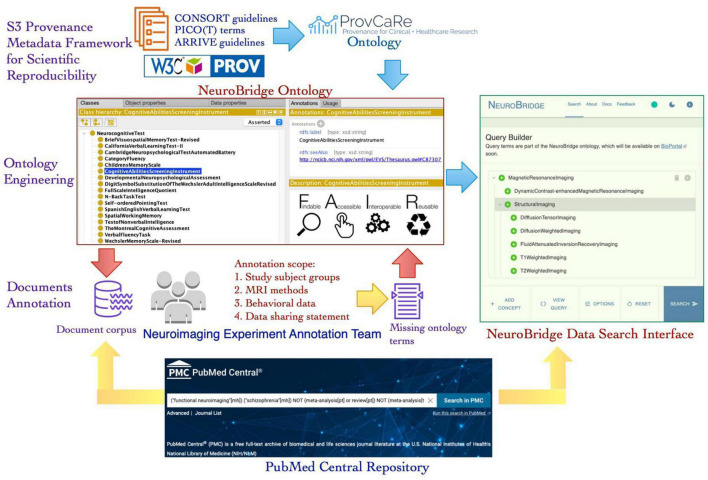
An overview of the new ontology engineering workflow developed in the NeuroBridge project to create a FAIR provenance ontology for neuroimaging experiments. The NeuroBridge ontology is based on the ProvCaRe S3 framework for metadata to support FAIR principles and it is used in annotation of full text articles retrieved from PubMed Central repository.

The NeuroBridge project aims to generalize and expand the SchizConnect platform to develop a data discovery system that can be a bridge between the needs of neuroimaging researchers and the relevant data from scientific literature. Published articles describing neuroimaging studies and datasets generated in these studies are an important resource for investigators. The NeuroBridge platform aims to automatically extract provenance metadata terms from these articles and use the terms to identify datasets that are highly relevant to a user’s research question. In this paper, we describe a novel iterative ontology engineering process that was developed and implemented to create the NeuroBridge ontology that supports: (1) Fine granularity annotation of full text articles describing neuroimaging studies; (2) Automated parsing and indexing of terms describing experimental design details of neuroimaging studies; and (3) Interactive user queries to locate experimental studies that match research terms (Figure 1). The automated parsing and indexing of research papers as well as the interactive user queries require the development of machine learning algorithms and web application resources together with the NeuroBridge ontology, therefore, they are outside the scope of this paper and are described in the companion paper (Wang et al., Under Review). The rest of the paper is structured as follows: In the Section “2. Materials and methods,” we describe the core components of the NeuroBridge ontology development process for text annotation; In the Section “3. Results,” we describe the resulting neuroimaging metadata ontology and its use in annotation of published literature; and in Section “4. Discussion and conclusion,” we discuss the broader impact of the NeuroBridge ontology engineering process, the terms of the ontology, and its application in making neuroimaging studies FAIR.

## 2. Materials and methods

The first phase of the ontology engineering process involved defining the scope of the ontology to support FAIR guidelines in the neuroimaging domain. Given the lack of existing community standards for modeling neuroimaging metadata, we built on our experience in dataset sharing efforts in the SchizConnect project (e.g., subject groups, neuroimaging modalities, cognitive and clinical assessments), and extended them to current literature describing substance abuse disorders studies using neuroimaging studies.

In the second phase, the metadata terms were classified into the three ProvCaRe S3 model categories of study data, instruments, and method. These metadata terms were collaboratively modeled in the NeuroBridge ontology and subsequently used to annotate full text articles describing neuroimaging experiments as part of the third phase of the ontology engineering process. In the final phase, the feedback from the metadata annotation phase was used to evaluate the NeuroBridge ontology followed by extensive restructuring and expansion to meet FAIR guidelines for neuroimaging datasets. Figure 1 is an overview of the new ontology engineering process developed in this project to model computable provenance metadata for neuroimaging experiments.

### 2.1. Document corpus describing neuroimaging experiments

We created a document corpus consisting of articles describing potential fMRI datasets generated from schizophrenia related studies by querying the PubMed Central repository for papers published between 2017 and 2020 using the following phrases:

Query 1: (”functional neuroimaging”[mh]) (”schizophrenia”[mh]) NOT (meta-analysis[pt] or review[pt]) NOT (meta-analysis[ti] or review[ti])

Similarly, the following query expanded on the above query with a focus on substance abuse aspect:

Query 2: (”functional neuroimaging”[mh]) (”substance-related disorders”[mh]) NOT (meta-analysis[pt] or review[pt]) NOT (meta-analysis[ti] or review[ti])

The first query expression generated a corpus consisting of 255 articles, while the second query expression generated 200 articles. We selected 100 articles from each query result to manually process and annotate them using provenance metadata terms. During the annotation phase, we removed articles that were reviews, or meta-analyses, or position papers related to the neuroimaging domain, which resulted in a final count of 186 articles in the document corpus. This corpus included a few papers published on the psychosis datasets available through SchizConnect, but the entirety of the substance abuse papers, and majority of the schizophrenia papers were not part of the SchizConnect project.

### 2.2. Modeling neuroimaging metadata terms in the NeuroBridge ontology

The W3C PROV specifications support the modeling of provenance metadata for multiple applications, including the description of how datasets were generated to enable their meaningful use (secondary use), reproducibility, and ensuring data quality (Lebo et al., 2013). To achieve these objectives, the PROV model consists of *prov:Entity*, which may be physical or digital (e.g., fMRI images), *prov:Activity* to model the process of creation or modification of entities (e.g., imaging protocol), and *prov:Agent*, which takes responsibility for an activity (e.g., study participant). In addition to these terms, the PROV specifications also includes relationships that can be used to represent detailed provenance metadata, for example an experimental study *prov:used* a neurocognitive test of language function [we refer to the PROV specification for further details (Moreau and Missier, 2013)]. The PROV ontology standardized these provenance metadata terms and relationships using OWL expressions. The PROV ontology was extended in the ProvCaRe ontology to standardize the S3 model (Sahoo et al., 2019).

**FIGURE 2:**
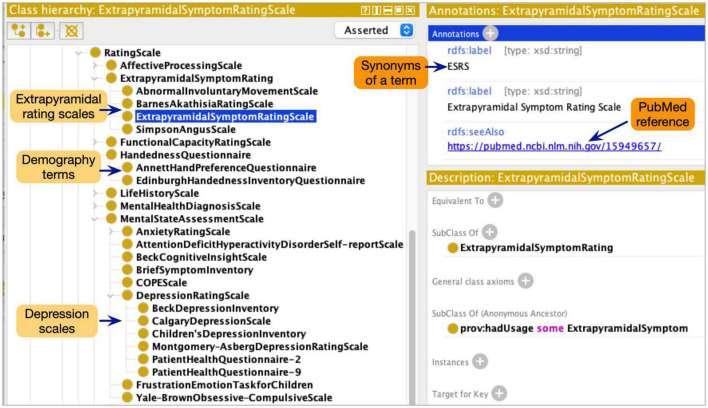
The NeuroBridge ontology models a variety of terminology collected as part of the previous SchizConnect project to describe schizophrenia related studies.

Although the ProvCaRe ontology models the core provenance metadata terms associated with biomedical health domain, the ontology does not model terms at the required level of granularity for neuroimaging experiments. Therefore, the NeuroBridge ontology restructured and expanded the ProvCaRe ontology with a focus on neuroimaging experiments and broadly neuroscience research studies. Our approach is based on ontology engineering best practices to re-use and expand existing ontologies for specific domain applications (Bodenreider and Stevens, 2006). In the initial phase of the ontology engineering process, we reviewed many existing neuroimaging terminologies to identify suitable terms for inclusion in the NeuroBridge ontology. First, we reviewed the SchizConnect terminology list that describes: (1) demography (e.g., socioeconomic status, and handedness scales questions in Edinburgh inventory rating scale among others); (2) psychopathology symptoms (e.g., Calgary depression scale, and Young mania rating scale); (3) extrapyramidal symptoms (e.g., Abnormal involuntary movement scale); (4) functional capacity (e.g., history of motor skills); and (5) medical condition (e.g., Structured clinical interview for the diagnostic statistical manual of mental disorder, SCID) (Spitzer et al., 1990). These five categories of SchizConnect terms were modeled as subtypes of different rating scales in the NeuroBridge ontology (Figure 2 shows a screenshot of the ontology class hierarchy representing these terms).

In the next step, we reviewed the Neuroimaging Data Model-Experiment (NIDM-E) ontology that was designed to describe different modalities of neuroscience datasets, including terms from the Digital Imaging and Communications in Medicine (DICOM) and Brain Imaging Data Structure (BIDS) specifications (Gorgolewski et al., 2016). We focused on mapping NIDM-E ontology terms describing the method used to generate neuroimaging data and its application in the NeuroBridge ontology. This process was facilitated by collaborative meetings with members of the NIDM-E team members to coordinate the reuse and mapping of terms between the two ontologies. In addition to NIDM-E ontology, we also used the National Center for Biomedical Ontologies (NCBO) BioPortal resource to create mappings between the NeuroBridge ontology and existing ontologies, such as the Radiology Lexicon (RadLex), the Systematized Nomenclature of Medicine Clinical Terms (SNOMED CT), and the National Institute on Drug Abuse (NIDA) common data elements (CDE). The NIDA Clinical Trials Network (CTN) recommended CDEs are part of the National Cancer Institute Data Standards Repository (caDSR), which were created using the metadata registry standard (ISO/IEC 11179) (National Institutes of Health, 2023).

At the end of this first phase of the ontology engineering process, the NeuroBridge ontology had a broad representation of provenance metadata terms describing neuroimaging studies. To evaluate the coverage of the NeuroBridge ontology, we used it for manual annotation of full text articles in our document corpus.

### 2.3. A two-pass process for text annotation using provenance metadata terms

In the next phase, we implemented a two-pass text annotation process that was designed to be *repeatable*, which could be used to annotate new metadata features in papers as they are identified, and *extensible*, which could be customized for annotation of experimental studies described in broader neuroscience articles. The first “draft expansion” pass was marked by extensive collaboration between the members of the text annotation and the ontology engineering team. The goal of this pass was to identify metadata terms that were needed for annotation of the papers, but they were missing in the first version of the ontology.

This phase was implemented using a variety of online tools, including spreadsheets and shared copies of published articles from the document corpus that were distributed using Google Drive. There were two main workspaces: the first workspace, implemented as a spreadsheet, listed the assignment of annotation team members to specific documents (two annotators per document), which recorded the citation, links to the documents, basic bibliographic data, and notations related to the annotation process, such as the agreement between annotators regarding the metadata annotations. The annotation team members were trained remotely via teleconference due to the coronavirus pandemic. The original team of annotators were trained by co-author JAT to find the relevant parts of papers for annotation.

After this training phase was completed, the annotation teams (with at least two members) were assigned the articles for annotation. A second workspace contained the annotations made by the annotation team members, with each row of this spreadsheet corresponding to a reviewed article and the metadata annotations listed in the columns. Both the workspaces were live documents that were modified by all the members of the annotation team.

The annotation team members focused on the title, abstract, and methods sections of the papers. Their goal was to identify the available or needed labels for each article with four categories of provenance metadata:

1. **Subject groups:** This included disorder types (e.g., schizophrenia, substance abuse) as well as control groups (identified as “no known disorder”) if present in the article.
2. **Imaging methods used in the study:** For example, resting state or task-based functional imaging, and T1 weighted imaging.
3. **Behavioral data collected in the study**. For example, standardized scales for symptom severity, cognitive batteries, personality assessments. In addition, unique non-standardized scales, and measures such as medication status or specific cognitive experiment data were also identified and annotated.
4. **Data and resource sharing**. Mark the presence or absence of a formal data sharing statement for the project.

The second workspace was used to record the above four categories of provenance metadata annotations associated with specific sections of text in the article. Additional columns in this workspace were used to record the agreement between members of the annotation team regarding the category of metadata terms. The annotators also used this workspace to record metadata terms that could not be mapped to an appropriate ontology term. These missing terms in the ontology together with feedback related to class structure of the ontology were used as feedback by the ontology engineering team to revise the NeuroBridge ontology.

### 2.4. Revision of the NeuroBridge ontology using text annotation feedback

As part of the tightly coupled cycle of ontology engineering and text annotation, the ontology engineering team agreed that no existing ontology terms were to be removed to preserve backward compatibility with metadata terms already used to annotate the articles. However, the annotations could be modified after the expanded version of the ontology was finalized. The feedback from the annotation phase identified missing terms across all the four categories of provenance metadata, that is, *subject groups*, *imaging methods*, *behavioral assessments*, and *data sharing* policy description.

Within the subject groups category, the ontology engineering team (co-authors, SSS, JAT, and LW), reviewed the modeling approach for representing the distinction between samples of unaffected family members of a study subject with a particular disorder, and “healthy controls.” We had already identified that “healthy controls” in any given study may or may not be defined in a consistent manner across studies; therefore, these terms were annotated as a group with “no known disorder” (the corresponding ontology term *NeuroBridge:NoKnownDisorder* was modeled as subclass of *NeuroBridge:ClinicalFinding*). This modeling approach allowed us to represent the information that these participants did not have the given disorder that characterized the other samples in the same study, but there was no guarantee they did not have some other disorder. It is important to note that in disorders with genetic risk, the relatives of affected individuals are of special interest. However, we deferred modeling this provenance information to the next version of the ontology as it required the annotation of a new set of articles describing whether family members of subjects are included in the “no known disorder” group, and the complexity of a family tree (sibling, parents, and multiple generations, among other terms). If the family members were not reported to have been diagnosed with any disorders, the annotation noted that the study collected the “no known disorder” subject group.

**FIGURE 3:**
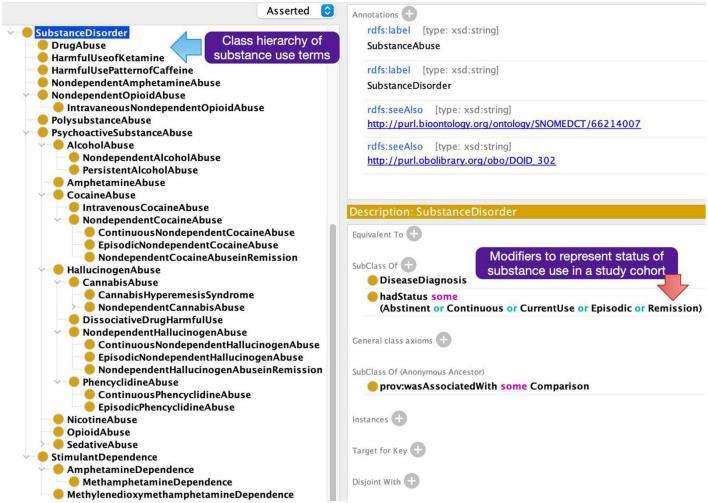
Additional neuroimaging metadata terms added to the expanded NeuroBridge ontology based on feedback from the annotation phase.

A particular challenge in annotating the articles with subject group metadata terms was the need to model modifying attributes of the descriptors in the NeuroBridge ontology. For example, the diagnosis label was not sufficient as a growing number of papers explicitly included subjects with the first episode of psychosis versus subjects with chronic schizophrenia, and unmedicated or medicated status of the study subject. Further, an important distinction in substance use research was not only the type of substance being used but also the “current status of use”; for example, it is important to distinguish between “currently abstinent users,” “currently dependent users,” and “children of people with addiction.” In the NeuroBridge ontology, we represented these through conjunctions of labels, “unmedicated and schizophrenia,” “currently abstinent,” and “currently using” (Figure 3).

**FIGURE 4:**
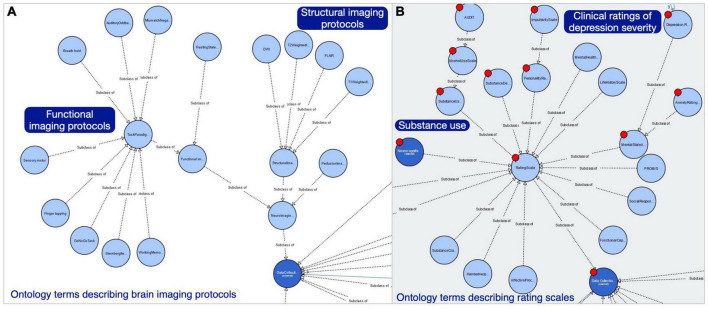
Expanded NeuroBridge ontology class structure for **(A)** imaging protocols, and **(B)** rating scales metadata terms used to describe neuroimaging experimental studies.

Similarly, we added new classes to the NeuroBridge ontology to distinguish between imaging protocol, and the imaging modality of the data collected by the imaging protocol. Within both the modality and the protocol branches of the ontology classes, there are common terms describing the types of structural and functional imaging, including task-based and resting state fMRI. The annotation team identified 30 unique terms to describe fMRI tasks in the article corpus. Nine of these terms had been modeled in the CogPO, which had been included in the NeuroBridge ontology. Figure 4A shows the ontology classes describing imaging protocols, which were modeled separately from the imaging modalities. In addition, the NeuroBridge ontology was expanded to model terms describing clinical symptom assessments, diagnostic interviews, and neuropsychological (cognitive) tests. Within the substance use disorder literature, however, there is a research effort focused on impulsivity’s relationships with addictive behavior, as well as measures of emotion regulation or openness or other personality traits. We created an initial branch in the ontology for personality assessments as well, to capture those measures. A subset of the Rating Scales is shown in Figure 4B showing (starting in the upper left) the AUDIT scale as an example of the Alcohol Use Scale, which is a type of Substance Use Scale; Substance Craving scales are a separate branch. Neurocognitive scales are not expanded in this view but include various cognitive batteries. The Barratt Impulsivity Scale (not shown in Figure 4) would be an Impulsivity scale class as a subclass of Personality Assessments (top). Clinical ratings of Depression severity (far right) are examples of Mental State Assessments, which are distinct from scales primarily used for diagnosis (modeled as subclasses of the Mental Health Diagnosis Scale class).

### 2.5. Final annotation phase of the document corpus

Following the first annotation pass through the corpus, and the extensions to the ontology that it entailed as discussed above, the second pass of annotations had a twofold goal of: (a) generating high quality, manually annotated text describing neuroimaging experiments, which were subsequently used to train a Bidirectional Encoder Representations from Transformers (BERT) deep learning model (Devlin et al., 2019; Wang et al., 2022); and (b) validate the metadata term coverage of the NeuroBridge ontology.

To achieve these two goals, an independent set of annotators used the *Inception* text annotation tool to confirm that the annotations originally marked in the spreadsheets could be used in annotating the text (Klie et al., 2018). The Inception tool allows users to select text spans (individual words or phrases) and then connect these spans to terms in the ontology. We used the revised version of the NeuroBridge ontology in this annotation pass (we note that the structure of the ontology remained unchanged during this pass). The annotation team members consisted of trained annotators and a curator. The curator had a supervisory role during the annotation process, specifically with the authority to make unilateral decisions in the annotation process. Curators reviewed the work of the annotators and resolved differences in their joint annotations, as well as reviewed all the annotations, and played an important role in ensuring consistency in term usage and application across the corpus.

**FIGURE 5:**
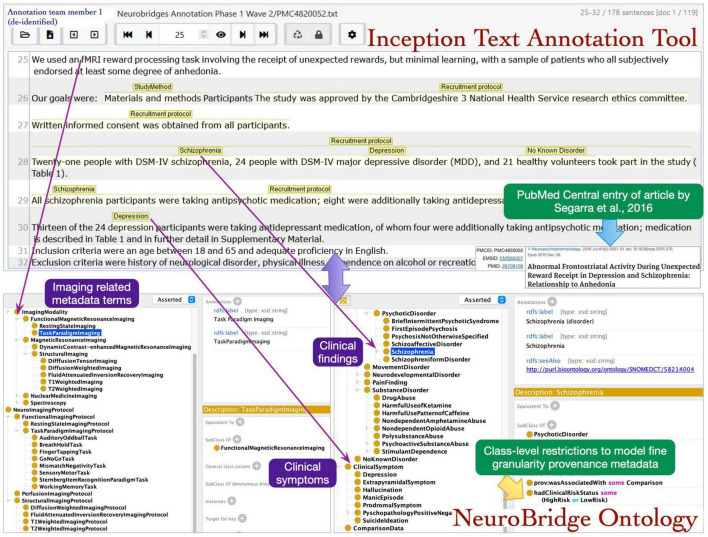
The annotation team members identified the text spans in articles during review and mapped the terms to NeuroBridge ontology classes.

The roles of annotator and curator were separated, with one of the annotators from the first annotation pass now serving as a curator, and new annotation team members were recruited for this annotation pass. The annotation process was implemented in the following steps: during step 1, a pair of annotators were assigned to an article. The annotators had access to the annotations in the spreadsheets from the first pass, which alerted them to the presence of expected metadata labels in each article. In step 2, each annotator individually reviewed the assigned article and using the annotation from first phase as a guide applied the final metadata annotations. Any Issues identified during this phase were reviewed by the supervisor. The annotators selected the spans of text representing metadata terms describing neuroimaging experiments and marked these with appropriate links to the ontology terms (Figure 5).

After each pair of annotators marked their work as complete, the papers were reviewed by a curator. The Inception software has a curator view of each document that allows direct comparison of the work of each assigned annotator. When the annotators agree completely, the curator can simply mark the annotations as correct or incorrect. When annotators disagree, the curator can decide how to resolve any differences in the final document. Initially the curator was the annotation supervisor. However, at this point some of the more senior annotators from the previous pass had developed sufficient skill; therefore, they were designated as curators for this phase. This allowed volunteer annotators, who had gained significant experience and knowledge about provenance metadata, to move onto a different category of annotation task.

The inter-annotator agreement was computed for the annotations done in Inception; the initial work that used online spreadsheets required the annotators to work in pairs to identify the terms needed for the ontology expansion, so agreement would not be meaningful. Inception calculated Cohen’s kappa as measures of pair-wise agreement between annotators, which ranged from 0.75 to 1.0 (mean 0.92). We exported the annotated text corpus from the Inception tool as *WebAnno TSV 3.x* files (this NeuroBridge resource^1^).

## 3. Results

The new iterative ontology engineering process implemented in this paper resulted in the first release version of the NeuroBridge ontology, consisting of more than 660 classes and 3,200 axioms representing a variety of neuroimaging experiment related provenance metadata. The ontology class expressions leverage more than 40 OWL object properties together with class level restrictions to represent the four categories of metadata information used during the annotation phase of this study. The ontology was evaluated using the Protégé built-in FaCT++ reasoner, which performed classification of concepts using subsumption reasoning followed by satisfiability to identify incorrect subsumptions (Tsarkov and Horrocks, 2006). The standard inference results computed by the reasoner across class, object property, and data property hierarchies as well as class, and object property assertions did not identify any errors in the ontology. The NeuroBridge ontology is made available at the National Center for Biomedical Ontologies (NCBO) Bioportal, as https://bioportal.bioontology.org/ontologies/NEUROBRG.

### 3.1. Provenance metadata terms used to annotate the document corpus

The 186 articles in the document corpus included annotations with 153 unique metadata terms. The annotation team used the metadata terms to label the study method text, the subject groups, the imaging techniques, and additional data. The annotation process ensured that there would be a minimum of one provenance metadata term for each of the first three of those categories, which resulted in a minimum number of concepts per article to be three, with the assumption that there was no other cognitive or behavioral data or information about the recruitment, which may occur with the use of legacy data. Table 1 shows the metadata annotations per paper (including repeats of the same annotation on different blocks of text) and the number of distinct metadata terms per paper. Given multiple imaging data types, multiple possible subject groups, and a wide range of assessments, the number of concepts annotated within the description of the study could range notably, as shown in Table 1.

**TABLE 1:** The descriptive statistics on the paper annotations.

	Annotations per paper	Concepts per paper
Minimum	5	3
Median	33	10
Mean	35	10
Maximum	84	21

Conversely, the number of papers referencing each concept ranged between 1 and the entire corpus; the median and average number of *papers per concept* were 3 and 12.54, respectively, representing a skewed distribution of papers referring to concepts. The most common concepts in this corpus are shown in Table 2. As expected, after Study Method and Recruitment Protocol, which is included in almost every study except some papers which used legacy data and gave no details, is the most common subject group (*NeuroBridge:NoKnownDisorder*), and the most common imaging techniques (*NeuroBridge:FunctionalMagneticResonanceImaging* and *NeuroBridge:T1WeightedImaging*). Disorders represented in these papers were chosen to include *NeuroBridge:schizophrenia*, which account for its common use; but substance use disorder was more diverse, with *NeuroBridge:AlcoholAbuse* and *NeuroBridge:CocaineAbuse* being the most common metadata terms.

**TABLE 2:** Concepts referred to in at least 10 papers, as well as their general superclass and the number of papers which referred to them.

Concept	Relevant superclass	Number of papers
StudyMethod	Activity	184
RecruitmentProtocol	StudyMethod	183
NoKnownDisorder	ClinicalFinding	154
FunctionalMagneticResonanceImaging	ImagingModality	128
T1WeightedImaging	StructuralImaging	99
MagneticResonanceImaging	ImagingModality	89
Schizophrenia	MentalDisorder/DiseaseDiagnosis	85
RestingStateImaging	FunctionalMagneticResonanceImaging	72
TaskParadigmImaging	FunctionalMagneticResonanceImaging	69
StructuredClinicalInterviewforDSMDisorders	RatingScale	61
MagneticResonanceImagingInstrument	ImagingInstrument	44
PositiveandNegativeSyndromeScale	RatingScale	44
StructuralImaging	ImagingModality	41
AlcoholAbuse	SubstanceDisorder	36
FunctionalImagingProtocol	BrainImaging	32
T2WeightedImaging	StructuralImaging	28
NeurocognitiveTest	RatingScale	26
SubstanceDisorder	DiseaseDiagnosis	21
AlcoholUseDisordersIdentificationTest	RatingScale	18
SchizoaffectiveDisorder	MentalDisorder/DiseaseDiagnosis	17
PsychoticDisorder	MentalDisorder/DiseaseDiagnosis	16
Questionnaire	DataCollectionInstrument	15
CocaineAbuse	SubstanceDisorder	15
FagerstromTestforNicotineDependence	RatingScale	14
ScaleforAssessmentofNegativeSymptoms	RatingScale	12
Electroencephalogram	DiagnosticProcedureOnBrain	12
MedicationStatus	ObservableMeasurement	12
BeckDepressionInventory	RatingScale	11
MentalHealthDiagnosisScale	RatingScale	11
NicotineAbuse	SubstanceDisorder	11
DrugDependence	DrugRelatedDisorder (SNOMED)	10
SubstanceUseScale	RatingScale	10
BipolarDisorder	MentalDisorder/DiseaseDiagnosis	10

Surprisingly, only 22% of the articles in our corpus of 186 recently published papers had an explicit data sharing and access statement, despite the increasing focus on data sharing within different domains of biomedical research. This statistic clearly highlights the challenges in making neuroimaging data findable and accessible.

### 3.2. Use of the ontology in the NeuroBridge user portal

In addition to its use in annotation of full-text articles, the NeuroBridge ontology also is incorporated into the NeuroBridge platform for use. The NeuroBridge platform allows users to compose a search query using ontology terms together with logical connectives such as AND, OR. The query expression is automatically expanded using OWL reasoning to include relevant subclasses of a selected ontology term, and this expanded query expression is used to search for neuroimaging experimental studies that match the query constraints (Hitzler et al., 2009). Please see our companion NeuroBridge paper in this Research Topic issue for more details of the platform (Wang et al., Under Review).

## 4. Discussion and conclusion

The NeuroBridge ontology combines the experience gained from neuroimaging data sharing projects, such as SchizConnect, NI-DM, CogPO and CogAtlas, with the S3 framework of the ProvCaRe project. This combination expands both ProvCaRe and the previous terminologies to capture important features of multiple domains of biomedical research. This positions NeuroBridge as a backbone for interoperability in annotating the neuroimaging literature. By incorporating substance use disorder papers in the corpus of this study, we confirmed that the S3 framework and the basic SchizConnect terminologies were sufficient to capture metadata information about neuroimaging studies in a different subfield of mental health. However, each of the different categories of metadata terms modeled in the ontology can be further extended to model additional study metadata describing its subject recruitment and data collection methods.

The metadata terms describing MRI techniques are similar across mental health studies and within our 186 functional imaging papers, 84 used task-based imaging, and the remaining 102 (55%) used resting state approaches. Within the task-based neuroimaging, there were a surprisingly limited number of tasks in these papers. The task name was not always specified in the text: For example, the Balloon Analogue Risk Task (BART) or the Monetary Incentive Delay Task were used in 15 of the papers, variations on a cue-reactivity paradigm were used in another nine papers, and the Stop Signal task in another seven papers for example. Naturalistic viewing was used in two papers, and another two dozen tasks such as reality monitoring, paced serial addition, or visual perspective taking were used once each in the corpus. A dozen papers did not include an explicitly named or recognizable imaging paradigm in the text. Future extensions of this corpus are planned to extend the representation of the task paradigms, and to annotate the descriptions of the task in the text. This would allow automated methods for text mining to group papers based on similar task descriptions, and to identify potential task labels that will be added to the ontology.

The rating scales and questionnaires used in the studies in this corpus cover a wide range of topics. We did not create labels for every scale identified in the annotation process. In the ontology, we classified the scales based on higher-level use, such as symptom severity ratings, personality scales, social function scales, and craving scales among others. This is not a challenging issue unique to neuroimaging study metadata, as every domain has its own clinical and cognitive tools, and new assessments and scales are developed continually. The NIH CDEs represent an effort to make data more interoperable, by representing common variables with standard terms. The NIMH National Data Archive (NDA) contains data from highly varied NIMH-funded studies across multiple experimental study designs and subject groups, all tagged with CDE terms. We explored using the NDA’s CDEs and matching those against the terms identified in the papers and incorporating them into the ontology, as the data archive is representative of recent research techniques. But there are several notable challenges to that approach, for example the CDEs do not have standardized structure which can be modeled as computable metadata terms. The CDE terms describe specific questions based on the studies that submit them. This can lead to idiosyncratic effects, for example, the term for the Scale for the Assessment of Positive Symptoms (SAPS) is defined as only the formal thought disorder symptom severity part of the SAPS, linked to psychiatric outcomes in Parkinson’s Disease, rather than being defined as the Scale itself. These limitations in terms of standardization and lack of structure excluded the NIH CDE for these scales from being modeled in the ontology.

We note that this study successfully demonstrated the implementation of an internationally coordinated metadata annotation process, and online annotation efforts of neuroimaging papers across multiple naive teams. Teams were recruited from several undergraduate programs in the US and in India, and students worked for research credit or in some cases for a summer stipend. The use of current distributed-access tools allowed interactions across teams, levels of expertise, and time zones.

### 4.1. Ontology-based data access and the application of NeuroBridge ontology

Searching for relevant information over a large corpus is challenging and this task is more difficult if the objective is to query information described in the article’s text. In this scenario, exact matches between query term and terms in text are difficult; therefore, deeper domain knowledge in the form of an ontology has been acknowledged to be an effective approach for processing unstructured text in the knowledge representation and Semantic Web communities. The main contribution in our approach is the use of the NeuroBridge ontology in the NeuroBridge search over published articles. The NeuroBridge search feature is designed to use machine learning techniques together with ontologies to support queries beyond simple syntactic and grammar-based term matching; it is designed to use multi-faceted ontology structure to perform domain-specific search. This captures the nuances of data references without being tied down to any specific syntactic structure.

The NeuroBridge ontology and the NeuroBridge platform are distinct from traditional systems such as the Ontology Based Data Access (OBDA) (also called Ontology Mediated or Ontology Based Query Answering) (OMQA/OBQA) (Kock-Schoppenhauer et al., 2017; Xiao et al., 2018; Corcho et al., 2020; Franco et al., 2020; Pankowski, 2021), which are mostly based on relational databases, either across a single database or federated databases with related schemas. The NeuroBridge model can be viewed as a reverse of the mapping advocated by OBDA systems. In our approach we look at ontologies as providing the entities in a database schema and map these ontological structures to sentences or groups of sentences in published articles. This reverse mapping allows us to find references to datasets of interest in an article. This reverse mapping from articles to ontologies is facilitated through the human-annotated stage where identification of relevant sentence structure is performed. These manually annotated examples are used to train machine learning (ML) model to identify similar mappings [described in our companion paper (Wang et al., Under Review)].

### 4.2. Limitations

A key limitation of this study is the use of a time-intensive ontology engineering process, which makes it challenging to scale the NeuroBridge ontology to include other domains such as cardiac or spinal imaging studies, or even brain tumor scanning. This would require novel expansion methods to be implemented to add new terms in the ontology. As noted above, we do not explicitly model all published assessments, and the model of subject groups, as currently implemented, does not capture all possibilities. We also have not modeled all the possible details of a neuroimaging study, for example, imaging protocol parameters, quality assurance steps, data processing and analysis phases together with their many parameters, and the statistical results or their interpretation. This version of the ontology would not support, for example, searching for datasets of a certain sample size, which used a particular MRI platform machine, or user queries based on their conclusions (e.g., searching for datasets which were used to support a certain hypothesis).

The representation of neuroimaging behavioral tasks in the ontology does not include the Cognitive Paradigm Ontology (CogPO) approach, which focused on describing the choice of stimuli, the instructions given to the subject, and the responses that the subjects were expected to make till now (Turner and Laird, 2012). The CogPO approach would be more detailed, and would allow disambiguation between, for example, studies which claimed the same type of task but used different stimuli, or between studies which used different names for the same task. This level of detail was considered to be outside of the scope for the first version of the ontology; therefore, it will be part of future expansion of the ontology.

## 5. Conclusion

The goal of this project is to apply metadata annotations which address FAIR guidelines to the literature of published human neuroimaging studies, even though the studies themselves may not be sharing their datasets through FAIR-compliant methods. The objective of the study is to meet an important requirement of making neuroimaging metadata computable through the NeuroBridge ontology, which will enable neuroimaging data to be compliant with the FAIR guidelines. The use of the ontology for text annotation and supporting user queries in the NeuroBridge portal will allow us to identify and present the relevant neuroimaging papers to the user and to request access from the study authors as necessary for re-use of experimental data. For the purposes of finding neuroimaging datasets that use similar methods and that could be aggregated for a novel analysis, the NeuroBridge ontology is addressing what current ontologies in the fields today are lacking, i.e., describing the methods that neuroimaging studies employed to collect the data. The NeuroBridge ontology is available at https://neurobridges.org/, and in BioPortal (Musen et al., 2012). The corpus will be available on the NeuroBridge website as well for re-use by the community (see text footnote 1). The NeuroBridge platform has been submitted to rrids.org for consideration for a research resource identifier.

## Data availability statement

The original contributions presented in this study are publicly available. The NeuroBridge ontology data is available through the NCBO Bioportal as https://bioportal.bioontology.org/ontologies/NEUROBRG and the annotated text will be available through https://github.com/NeuroBridge/Annotation-Project/releases.

## Author contributions

SS, LW, MT, and JT conceptualized and designed the study. JT, MT, AA, and YW developed the annotation process involving the identification of metadata terms and their use in annotation of neuroimaging articles, with input from HL. SS, LW, and JT revised and developed the ontology with input from JA, AA, HL, AR, MT, and YW. JT, MT, and SS co-wrote the first draft of the manuscript. All authors contributed to the manuscript revision and read and approved the submitted version of the manuscript.

## Footnotes

1
https://github.com/NeuroBridge/Annotation-Project/releases
